# Supplementary material for: Distinct and separable roles for EZH2 in neurogenic astroglia
Source: eLife. 2014 May 27;3:e02439. doi: 10.7554/eLife.02439 (PMC4032491; doi:10.7554/eLife.02439)
Supplement: Supplementary file 1. — Quantity and dilution ratios of the primary antibodies used in this study. DOI: http://dx.doi.org/10.7554/eLife.02439.019 [file elife02439s001.docx]

| **Antibodies used for ChIP** | |  |  |  |
| --- | --- | --- | --- | --- |
|  |  |  |  |  |
| **Antibody** | **Type** | **Company/Source** | **Catalog #** | **Amount** |
| H3K4me3 | Rabbit Polyclonal | Active Motif | 39159 | 2µg/IP |
| H3K27me3 | Rabbit Polyclonal | Millipore | 07449 | 2µg/IP |
| EZH2 (AC22) | Mouse Monoclonal | Active Motif | 39875 | 2µg/IP |
| IgG | Rabbit | Millipore | PP64B |  |
|  |  |  |  |  |
| **Antibodies used for ICC/IHC** | |  |  |  |
|  |  |  |  |  |
| **Antibody** | **Type** | **Company/Source** | **Catalog #** | **Dilution** |
| BrdU | Rat monoclonal | Abcam | Ab6326 | 1:500 |
| DCX | Rabbit Polyclonal | Abcam | ab18723 | 1:1000 |
| DCX | Guinea Pig Polyclonal | Millipore | ab2253 | 1:500 |
| DLX2 | Guinea Pig Polyclonal | Gift from Yoshikawa lab |  | 1:2000-4000 |
| EZH2 | Mouse Monoclonal | BD | 612666 | 1:500 |
| GFAP | Chicken Polyclonal | Abcam | Ab4674 | 1:500 |
| GFAP | Rabbit Polyclonal | DakoCytomation | z0334 | 1:500 |
| GFP | Chicken Polyclonal | abcam | ab13970 | 1:500 |
| H3K27me3 | Rabbit Polyclonal | Millipore | 07449 | 1:500 |
| Ki67 | Rabbit Polyclonal | Abcam | ab16667 | 1:500 |
| NeuN | Chicken Polyclonal | Millipore | ABN91 | 1:500 |
| OLIG2 | Rabbit Polyclonal | Millipore | ab9610 | 1:500 |
| S100b | Rabbit Polyclonal | DakoCytomation | Z0311 | 1:500 |
| Tuj1 | Mouse Monoclonal | covance | MMS-435P | 1:1000 |
| Vimentin | Chicken Polyclonal | Millipore | ab5733 | 1:500 |
|  |  |  |  |  |

**Supplemental Table 1. Quantity and dilution ratios of the primary antibodies used in this study.**
