# Supplementary material for: Distinct and separable roles for EZH2 in neurogenic astroglia
Source: eLife. 2014 May 27;3:e02439. doi: 10.7554/eLife.02439 (PMC4032491; doi:10.7554/eLife.02439)
Supplement: Supplementary file 2. — Primers used for ChIP-qPCR experiments in this study. DOI: http://dx.doi.org/10.7554/eLife.02439.020 [file elife02439s002.docx]

| **Primers used for ChIP-qPCR** | |  |  |  |
| --- | --- | --- | --- | --- |
| *HoxA2b* Forward | AAGTTGATGGCGAAGGAAGA |  |  |  |
| *HoxA2b* Reverse | GCCTCTCGCCTCCTCTTAAT |  |  |  |
| *Dlx2* Promoter Forward | CGGGACAGGAAAGAGCAC |  |  |  |
| *Dlx2* Promoter Reverse | GAAGGGACCCGGAGAGATA |  |  |  |
| *Olig2* Promoter Forward | ACTAATGACTGCCTGGGTGTCT |  |  |  |
| *Olig2* Promoter Reverse | TCGCAACAGGAGTTATTGGA |  |  |  |
| *Gapdh* Promoter Forward | TCCCCTCCCCCTATCAGTTC |  |  |  |
| *Gapdh* Promoter Reverse | GACCCGCCTCATTTTTGAAA |  |  |  |
| *Arf* Promoter Forward | AAAACCCTCTCTTGGAGTGGG |  |  |  |
| *Arf* Promoter Reverse | GCAGGTTCTTGGTCACTGTGAG |  |  |  |
| *Arf* Downstream Forward | CTCCCTTTGCTACCCCTGAGAG |  |  |  |
| *Arf* Downstream Reverse | TTACTTATTTCGCTCCCATCCAC |  |  |  |
| *Ink4a* Promoter Forward | GATGGAGCCCGGACTACAGAAG |  |  |  |
| *Ink4a* Promoter Reverse | CTGTTTCAACGCCCAGCTCTC |  |  |  |

**Supplemental Table 2. Primers used for ChIP-qPCR experiments in this study.**
